# Seizure Outcomes and Predictors of Recurrent Post-Stroke Seizure: A Retrospective Observational Cohort Study

**DOI:** 10.1371/journal.pone.0136200

**Published:** 2015-08-26

**Authors:** Tomotaka Tanaka, Hiroshi Yamagami, Masafumi Ihara, Rie Motoyama, Kazuki Fukuma, Tetsuya Miyagi, Kazutaka Nishimura, Kazunori Toyoda, Kazuyuki Nagatsuka

**Affiliations:** 1 Department of Stroke and Cerebrovascular Diseases, Division of Neurology, Research Institute, National Cerebral and Cardiovascular Center, Osaka, Japan; 2 Department of Stroke and Cerebrovascular Diseases, Division of Cerebrovascular Medicine, Research Institute, National Cerebral and Cardiovascular Center, Osaka, Japan; Cleveland Clinic, UNITED STATES

## Abstract

**Background:**

Seizure is a common complication after stroke (termed “post-stroke seizure,” PSS). Although many studies have assessed outcomes and risk factors of PSS, no reliable predictors are currently available to determine PSS recurrence. We compared baseline clinical characteristics and post-stroke treatment regimens between recurrent and non-recurrent PSS patients to identify factors predictive of recurrence.

**Methods:**

Consecutive PSS patients admitted to our stroke center between January 2011 and July 2013 were monitored until February 2014 (median 357 days; IQR, 160–552) and retrospectively evaluated for baseline clinical characteristics and PSS recurrence. Cumulative recurrence rates at 90, 180, and 360 days post-stroke were estimated by Kaplan—Meier analysis. Independent predictors of recurrent PSS were identified by Cox proportional-hazards analysis.

**Results:**

A total of 104 patients (71 men; mean age, 72.1 ± 11.2 years) were analyzed. PSS recurred in 31 patients (30%) during the follow-up. Factors significantly associated with PSS recurrence by log-rank analysis included previous PSS, valproic acid (VPA) monotherapy, polytherapy with antiepileptic drugs (AEDs), frontal cortical lesion, and higher modified Rankin Scale score at discharge (all p < 0.05). Independent predictors of recurrent PSS were age <74 years (HR 2.38, 95% CI 1.02–5.90), VPA monotherapy (HR 3.86, 95% CI 1.30–12.62), and convulsions on admission (HR 3.87, 95% CI 1.35–12.76).

**Conclusions:**

Approximately one-third of PSS patients experienced seizure recurrence within one year. The predictors of recurrent PSS were younger age, presence of convulsions and VPA monotherapy. Our findings should be interpreted cautiously in countries where monotherapy with second-generation AEDs has been approved because this study was conducted while second-generation AEDs had not been officially approved for monotherapy in Japan.

## Introduction

Cerebrovascular disease is the most commonly identified cause of adult-onset epilepsy. Cardioembolic infarction, hemorrhagic stroke, and cortical lesions are most commonly associated with seizures, called post-stroke seizures (PSS).[1–6] In aged individuals, PSS accounts for 39%–45% of all seizures.[7–9] In general, PSS is divided into two categories, early and late seizures, according to the timing of PSS, which is distinguished by the cutoff time-point of two weeks.

However, early seizures typically occur within the first few days after stroke, whereas late seizures have a peak within 6 to 12 months with a higher frequency of up to 66.7% of stroke survivors.[1, 6, 10–12].

Recurrence of PSS is another clinical problem for stroke survivors. The recurrence risk of PSS appears higher in the case of late seizures than in the case of early seizures.[13] In one study, recurrent PSS developed in about 50% of patients who experienced late seizures but in only about 30% of patients with early seizures.[14] One observational study reported that about 50% of patients who received antiepileptic drugs (AEDs) after a first PSS episode had at least one recurrence during the follow-up period of 47 months.[15] Nevertheless, there are few reliable predictors of recurrent PSS. In addition, although many physicians prescribe AEDs for secondary prevention of PSS, it is uncertain which AEDs are most effective for the prevention of recurrence. Therefore, in this hospital-based retrospective study, we sought to identify reliable predictors of recurrent PSS by univariate and multivariate analyses in patients with late seizures and to evaluate the outcome of different AED regimens for secondary prevention of PSS.

## Methods

### Study Population

All patients admitted to our department of stroke and cerebrovascular diseases in National Cerebral and Cardiovascular Center (NCVC) were registered on a database (the NCVC Stroke Registry; Clinical Trials.gov: NCT02251665). NCVC is a specialized center and our department mainly treats acute stroke patients. Data for the registry were collected systematically at the time of discharge. From the data of 4325 patients admitted between January 2011 and July 2013 (cerebral infarction, 1576 patients; transient ischemic attack, 265; intracerebral hemorrhage, 574; and other neurological diseases including seizure, 1905), we identified consecutive patients diagnosed with seizure and selected patients with a history of preceding stroke. According to the epidemiological guidelines developed by the International League Against Epilepsy (ILAE), seizures occurring at least two weeks after stroke were defined as late seizures. Using this definition, we evaluated patients with late seizures for whom sufficient data were available in the patient records from hospitalization and outpatient clinic follow-up visits. We also distinguished post-stroke seizures (PSS) from post-stroke epilepsy according to seizure frequency after stroke: we defined PSS as single or multiple episode(s) after stroke and post-stroke epilepsy as recurrent seizures following stroke. The diagnosis of PSS was made by stroke neurologists based on electroencephalogram (EEG) findings and/or seizure semiology.

### Ethic Statement

The study was approved by the local ethics committee of National Cerebral and Cardiovascular Center. Oral consent for use of clinical records was taken from patients during follow-up. Written consent was not acquired because it is a retrospective study. All patients’ data were anonymized prior to analysis.

### Clinical Characteristics

The following demographic and clinical characteristics were collected from medical records: age, sex, previous stroke type (cerebral infarction, primary intracerebral hemorrhage, transient ischemic attack, or subarachnoid hemorrhage), previous late seizures, traumatic brain injury, neurosurgical intervention, dementia, family history of epilepsy, smoking, drinking alcohol, and stroke risk factors (hypertension, diabetes mellitus, dyslipidemia, and atrial fibrillation). Only prior history of late seizures was recorded from the medical database and documents in our hospital. Acute symptomatic seizure, early seizure after stroke, or other conditions known to lead to seizures were excluded.

Functional disability at discharge was evaluated by the modified Rankin Scale (mRS) from 0 (no symptoms) to 5 (severe disability). Scores were dichotomized as good (0–2) or poor (3–5).[16]

#### Clinical features and examinations

Seizure type was distinguished as focal, generalized, or unclassified according to ILAE guidelines. The presence of convulsions and postictal paralysis (Todd’s paralysis) was also confirmed. Magnetic resonance imaging (MRI) was performed in 94 patients (90.4%) using a 1.5-tesla MR unit (Magnetom Sonata or Magnetom Vision; Siemens Medical Solutions, Erlangen, Germany) within 24 h of PSS. Our routine MRI stroke protocol includes axial spin-echo diffusion-weighted imaging (DWI) (b = 0–1000 s/mm^2^) and axial fluid attenuated inversion recovery (FLAIR). The location of lesions was defined by areas of high signal intensity on DWI. The degree of white matter hyperintensity was graded semiquantitatively on FLAIR images by the modified Fazekas scale (0–3).[17] Lesion presence/location a+nd modified Fazekas scale scores were evaluated independently by at least three board-certified neurologists (among TT, KF, TM, RM, and KN). An interictal EEG was performed in 92 patients (88.5%) in the stroke unit. The EEG recordings were analyzed for focal or generalized slowing of background activity and paroxysmal activity.

### Follow-Up Assessment

The last follow-up visit, telephone interview, or the time of another recurrent seizure was considered the study termination point for each patient. We only counted seizures after the discharge from hospitals as a recurrence. Information regarding recurrent PSS was collected through inspection of inpatient and outpatient records or telephone inquiry with the patients themselves or relatives: the seizure recurrence, efficacy and modifications of AEDs and neurological status were recorded. Information on AEDs administered at the time of recurrence or at the end of the follow-up were also collected.

### Statistical analyses

Statistical analyses explored predictors of recurrent PSS. Differences in clinical and demographic variables between PSS and non-PSS patients were evaluated by univariate logistic regression. We used independent *t* tests or Mann—Whitney *U* tests for continuous variables and Pearson’s χ^2^ tests for categorical variables. Distributed variables are presented as mean ± standard deviation (SD) or medians (interquartile range, IQR). We also performed Kaplan—Meier analysis to determine cumulative recurrent PSS rates by 90 days, 180 days, and 1 year after the first episode of PSS, and cumulative rates were compared across patient groups using the log-rank test.

Cox proportional hazards regression models were used to evaluate independent predictors of recurrent PSS. The multivariate model included all parameters that had a P < 0.20 by log-rank test and the following pre-specified covariates: age (older or younger than 74 years), history of prior PSS, valproic acid (VPA) monotherapy, phenytoin (PHT) monotherapy, AED polytherapy, frontal cortical lesion, dyslipidemia, diabetes mellitus, atrial fibrillation, presence of convulsions, and mRS score (poor or good). Results are presented as hazard ratios with 95% confidence intervals (CIs). A *P* ≤ 0.05 is considered statistically significant. All statistical analyses were performed using the JMP 10 software package (SAS Institute Inc., Cary, NC, USA).

## Results

### Study population

**Fig 1:**
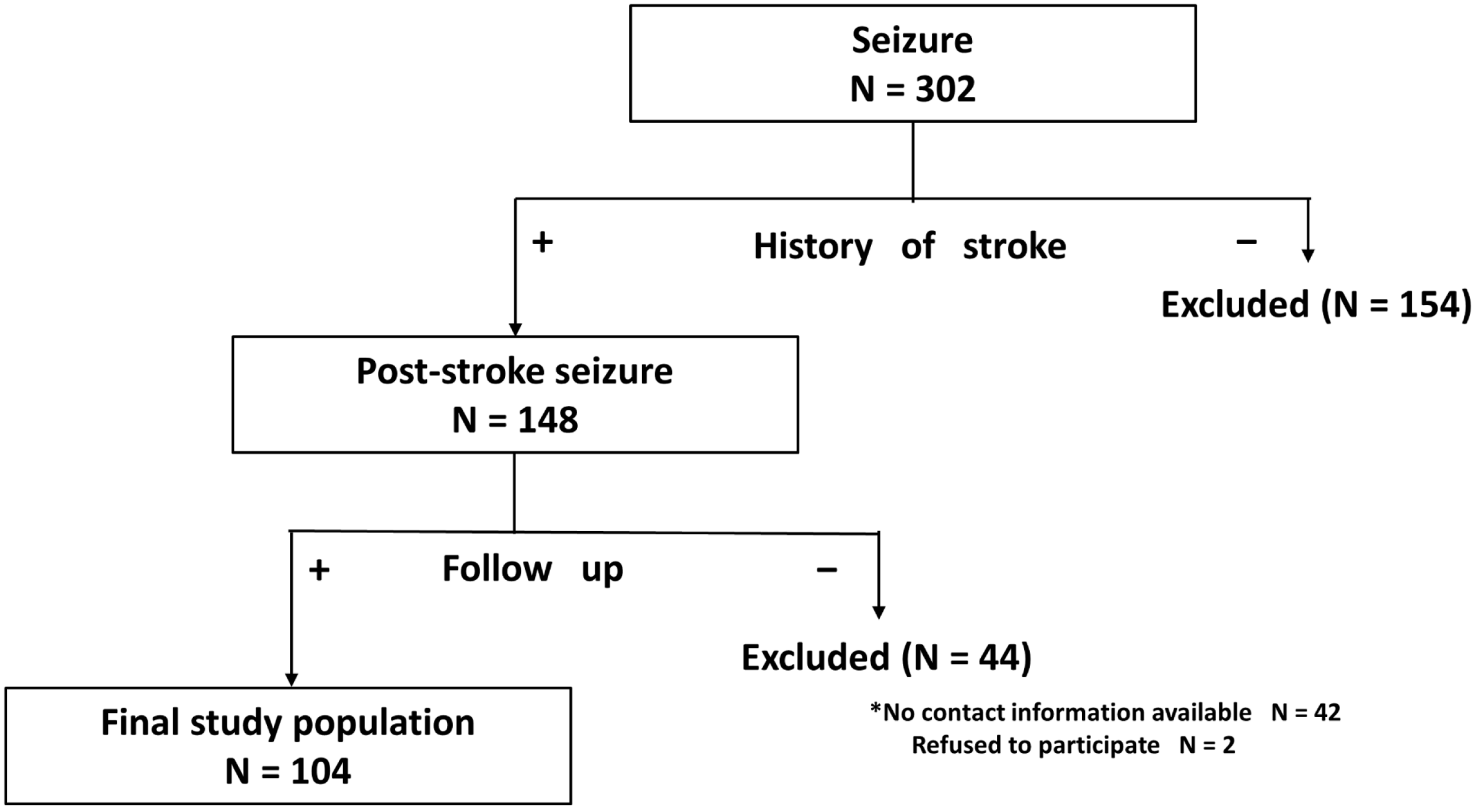
Study design and protocol. PSS, post-stroke seizures.

We identified 302 consecutive patients with seizures admitted to our department between January 2011 and July 2013. We excluded 154 patients who had no history of stroke and 44 patients for whom follow-up interviews or examinations could not be conducted for the following reasons: we could not contact the patients or relatives (n = 36), we could contact the patients or relatives but could not obtain sufficient data (n = 6), or the patients or relatives refused to participate in the study (n = 2). A total of 104 patients fulfilled our study criteria and were included in the analyses (Fig 1). Among the 104 patients, 97 patients (93.3%) were transported by ambulance with a suspicion of stroke or convulsions, and 63 patients (60.6%) had been treated in our hospital before the index admission.

Baseline demographic and clinical characteristics of the cohort are summarized in Table 1. The 71 men and 33 women had a median age of 74 years (interquartile range, 63.3–81.0 years). The median duration time from stroke to the index seizure was 1029 (IQR: 352–2626) days, except for 35 patients for whom the exact date of last stroke onset was not available. The stroke type was transient ischemic attack (TIA) in 3 patients (2.9%), cerebral infarction (CI) in 66 patients (63.5%), intracerebral hemorrhage (ICH) in 37 patients (35.6%), and subarachnoid hemorrhage (SAH) in 6 patients (5.8%) (Table 1). Some patients had two different episodes of stroke subtypes (CI and ICH, 7 patients; CI and SAH, 1 patient; CI and SAH, 2 patients). Many patients (71.9%) had a cortical lesion and 28 patients (26.9%) had atrial fibrillation. Seizure recurrence was found in 31 of the 104 patients, and 20 of the 31 patients (64.5%) had already experienced at least one episode of seizure when enrolled in this study. In addition, patients with no prior history of seizure (n = 52) were free of AED medication. However, among 52 patients with prior history of seizures, 23 patients were on AED monotherapy [VPA: 13 patients, PHT: 5, carbamazepine: 4, and zonisamide: 1], 9 patients were on AED polytherapy, and the other 20 patients were free of AED medication. In the total cohort of PSS patients, 77% had hypertension, 26% had diabetes mellitus, and 40% had dyslipidemia. On admission for PSS, 73 out of 104 patients (70.2%) exhibited partial seizures alone or with secondary generalization, 70 (67.3%) presented with convulsions, and 24 (23.1%) exhibited postictal (Todd’s) paralysis (Table 2).

**Table 1:** Comparison of baseline characteristics between post-stroke seizure patients with and without recurrence.

	Total (n = 104)	Recurrent PSS (n = 31)	No Recurrence (n = 73)	*p* value*
**Age, median (IQR), y**	74 (63.3–81)	71 (62–77)	75 (64.5–82)	0.17
**Male, n (%)**	71 (68.3)	22 (71.0)	49 (67.1)	0.70
**Follow up, median (IQR), day**	361.5 (172.3–551.8)	150 (93–316)	431 (302.5–659.5)	< .0001
**Time from stroke onset to the admission** ^§^ **, median (IQR), day**	1029 (352–2625.5)	749 (162–1348)	1056.5 (401–2792.3)	0.19
**TIA**	3 (2.9)	0 (0.0)	3 (4.1)	0.25
** Cerebral infarction**	66 (63.5)	19 (61.3)	47 (64.4)	0.76
** Intracerebral hemorrhage**	37 (35.6)	13 (41.9)	24 (32.9)	0.38
** Subarachnoid hemorrhage**	6 (5.8)	0 (0.0)	6 (8.2)	0.10
**Localization of lesion, n (%)**				
** Cortical**	69 (71.9)	24 (88.9)	45 (65.2)	0.020
** Frontal**	40 (38.5)	19 (61.3)	21 (28.8)	0.002
** Temporal**	40 (38.5)	12 (38.7)	28 (38.4)	0.97
** Parietal**	34 (32.7)	12 (38.7)	22 (30.1)	0.39
** Occipital**	17 (16.3)	5 (16.1)	12 (16.4)	0.97
**Traumatic brain injury, n (%)**	10 (9.6)	4 (23.5)	6 (13.0)	0.31
**Neurosurgical intervention, n (%)**	19 (18.3)	7 (22.6)	12 (16.4)	0.46
**Dementia, n (%)**	24 (23.1)	6 (19.4)	18 (24.7)	0.61
**Previous seizure** ^†^ **, n (%)**	52 (50.0)	20 (64.5)	32 (43.8)	0.05
**Familial seizure history, n (%)**	1 (1.4)	0 (0.0)	1 (2.0)	0.51
**Functional disability (mRS) at discharge, median (IQR)**	2 (0–4)	3 (1–4)	2 (0–4)	0.21
**Stroke risk factors, n (%)**				
** Hypertension**	80 (76.9)	26 (83.9)	54 (74.0)	0.27
** Diabetes mellitus**	27 (26.0)	4 (12.9)	23 (31.9)	0.044
** Dyslipidemia**	42 (40.4)	15 (48.4)	27 (37.0)	0.28
** Atrial fibrillation**	28 (26.9)	13 (41.9)	15 (21.4)	0.034
**Drinking alcohol, n (%)**	28 (26.9)	8 (27.6)	20 (30.3)	0.79
**Smoking, n (%)**	26 (25.0)	8 (27.6)	18 (27.7)	0.99

Data are expressed as median (IQR: interquartile range) or number of patients (%). Abbreviations: PSS, post-stroke seizures; TIA, transient ischemic attack; mRS, modified Rankin Scale.

* The Mann—Whitney *U* test was used to compare continuous variables and Pearson’s χ^2^ test to compare categorical variables.

^§^ The time calculated from the last stroke onset to the index (study entry) seizure, except for 35 patients for whom we could not obtain the exact date of last stroke onset.

^**†**^ Previous seizure: history of at least one episode of late seizure when enrolled in this study.

**Table 2:** Clinical features and examinations conducted on admission.

	Total (n = 104)	Recurrent PSS (n = 31)	No Recurrence (n = 73)	*p* value*
***Clinical features***				
** Seizure type, n (%)**				
** Generalized onset**	19 (18.3)	4 (12.9)	15 (20.6)	0.36
** Partial onset**	73 (70.2)	24 (77.4)	49 (67.1)	0.29
** Unclassifiable**	12 (11.5)	3 (9.7)	9 (12.3)	0.70
** Convulsions, n (%)**	70 (67.3)	25 (80.7)	45 (61.6)	0.06
** Postictal paralysis, n (%)**	24 (23.1)	8 (28.6)	16 (25.8)	0.78
***Examinations***				
** EEG findings** ^†^ **, n (%)**				
** Focal or lateralized slowing**	60 (65.2)	18 (72.0)	42 (82.4)	0.30
** Epileptiform discharge**	32 (32.6)	11 (36.7)	21 (33.9)	0.79
**MRI findings** ^‡^ **, n (%)**				
**DWI high intensity lesion after PSS**	29 (29.6)	8 (26.7)	21 (30.9)	0.67
**Modified Fazekas Scale, median (IQR)**	2 (1–3)	2 (1–3)	2 (1–3)	0.91
** Grade 0**	15 (16.0)	1 (3.3)	14 (21.9)	
** Grade 1**	20 (21.3)	11 (36.7)	9 (14.1)	
** Grade 2**	24 (25.5)	8 (26.7)	16 (25.0)	
** Grade 3**	35 (37.2)	10 (33.3)	25 (39.1)	

Data are expressed as median (IQR: interquartile range) or number of patients (%). Abbreviations: PSS, post-stroke seizures; EEG, electroencephalogram; MRI, magnetic resonance imaging; DWI, diffusion weighted imaging.

* The Mann—Whitney *U* test was used to compare continuous variables and Pearson’s χ^2^ test to compare categorical variables.

^**†**^ An interictal EEG was performed in 92 patients (88.5%).

^**‡**^ MRI was performed in 94 patients (90.4%).

### Incidence of recurrent PSS

**Fig 2:**
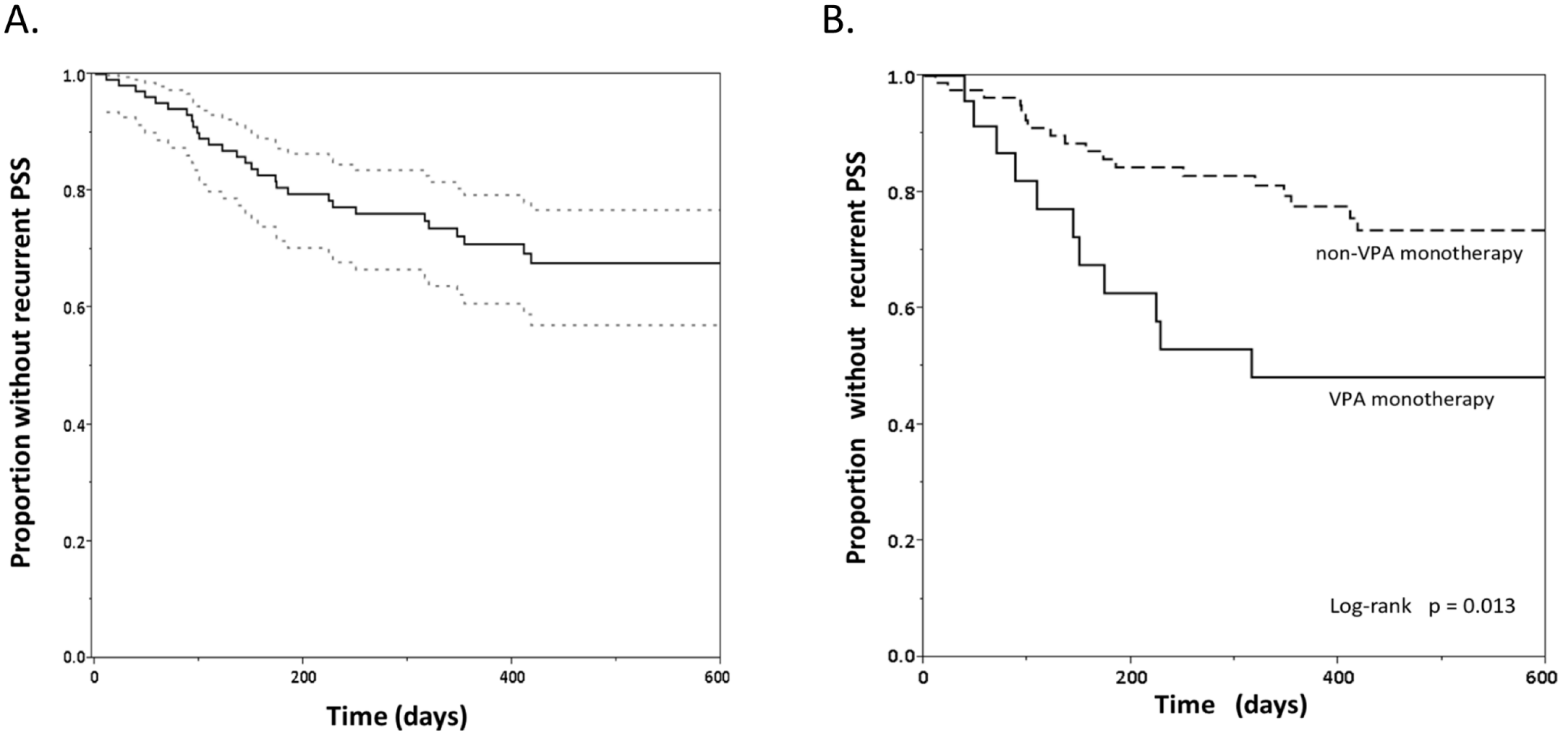
Kaplan—Meier curves for proportion without recurrent PSS. **A.** After a median follow-up of 362 days, we identified recurrent PSS in 31 patients (29.8%). Dotted lines indicate the confidence interval. **B.** For patients who received (solid line) or did not receive (dotted line) valproic acid (VPA) monotherapy at the time of recurrence or end of follow-up. VPA monotherapy may be insufficient to prevent recurrent PSS (log-rank test, p = 0.013).

After a median follow-up of 362 days (IQR: 172–552), we identified another recurrent PSS in 31 patients (29.8%). The cumulative proportions without recurrent PSS as estimated by Kaplan—Meier curves were 93.0% at 90 days (95% CI = 90.3–95.7), 80.6% at 180 days (95% CI = 76.6–84.6), and 70.9% at one year (95% CI = 66.1–75.7) (Fig 2). Among the 31 patients with recurrent PSS, only 2 patients had discontinued AEDs and 10 were taking two or more AEDs (polytherapy) at the time of recurrence (Table 3).

**Table 3:** Anti-epileptic drug regimens at the time of recurrence or end of follow-up.

	Total (n = 104)	Recurrent PSS (n = 31)	No Recurrence(n = 73)	*p* value*
**AEDs use, n (%)**				
** VPA monotherapy**	23 (22.1)	11 (35.5)	12 (16.4)	0.032
** CBZ monotherapy**	15 (14.4)	2 (6.5)	13 (17.8)	0.13
** PHT monotherapy**	22 (21.2)	4 (12.9)	18 (24.7)	0.18
** Other AED monotherapies** ^†^	10 (9.6)	2 (6.5)	8 (11.0)	0.48
** Polytherapy**	20 (19.2)	10 (32.3)	10 (13.7)	0.028
** None or discontinuation**	14 (13.5)	2 (6.45)	12 (16.4)	0.17

Data are expressed as median (IQR: interquartile range) or number of patients (%: percentages). Abbreviations: AEDs, antiepileptic drugs; PSS, post stroke seizures; VPA, valproic acid; CBZ, carbamazepine; PHT, phenytoin.

* The Mann—Whitney *U* test was used to compare AED regimens between recurrent PSS and no recurrent groups.

^†^Other AED monotherapies: 3 patients were taking zonisamide and 7 patients were taking levetiracetam.

### Predictors of recurrent PSS

The results of univariate analysis for all variables regarded as potential predictors of recurrent PSS are presented in Table 1. Patients with recurrent PSS were more likely to have a cortical lesion (especially in the frontal lobe), history of at least one episode of seizure when enrolled in this study, and atrial fibrillation, and they were less likely to have diabetes mellitus (p ≤ 0.05). There were no other differences in the baseline characteristics between patients with and without recurrence. Presence of high signal intensity on DWI or FLAIR images and slow waves or sharp and spike waves on EEG did not correlate with recurrent PSS. However, in the treatment before the recurrent PSS, recurrent PSS was more frequent in patients on VPA monotherapy or polytherapy than in patients on other single AEDs or no AED treatment (p ≤ 0.05) (Fig 2).

**Fig 3:**
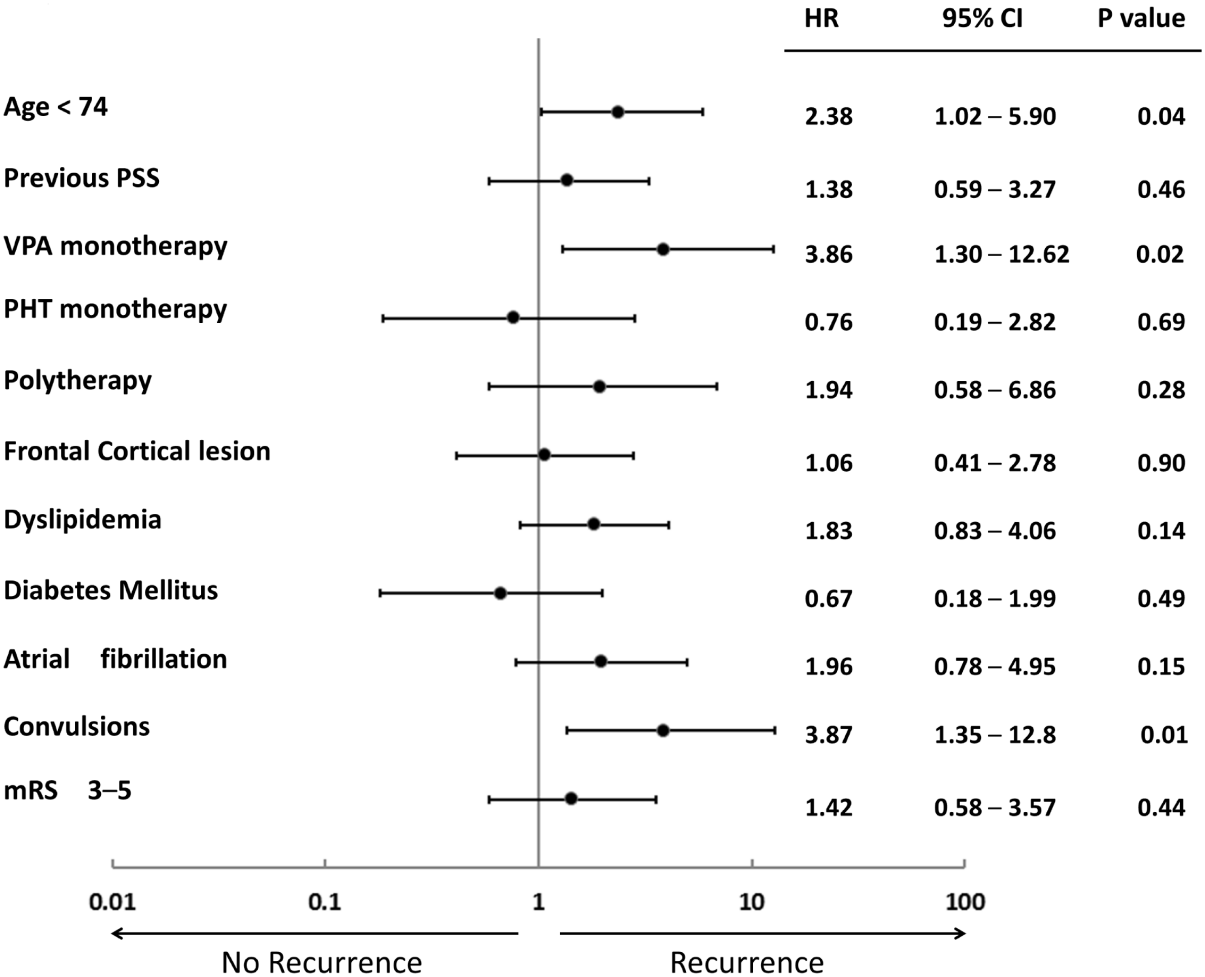
Association of designated factors with presence or absence of recurrent PSS. Hazard ratios (HR) (dots), 95% confidence intervals (95% CI) (error bars), and P values are from Cox proportional hazards models adjusted for age (<74 years), previous post-stroke seizures (PSS), valproic acid (VPA) monotherapy, phenytoin (PHT) monotherapy, polytherapy, frontal cortical lesion, dyslipidemia, diabetes mellitus, atrial fibrillation, presence of convulsions on admission, and poor modified Rankin Scale (mRS) score (3–5).

Of the 34 variables assessed by Kaplan—Meier methods (log-rank tests) to identify candidate predictors of recurrent PSS, younger age (<74 years), cortical lesion, frontal cortical lesion, at least one episode of seizure when enrolled in this study, poor mRS at discharge (3–5), presence of convulsions on admission, VPA monotherapy, and polytherapy were associated with recurrent PSS (p ≤ 0.05) (Table 4). In subsequent multivariable Cox proportional hazard model analysis including candidate predictors with p < 0.2 by log-rank analysis, three variables remained independently associated with recurrent PSS: younger age (<74 years) (hazard ratio, 2.38; 95% CI, 1.02–5.90), presence of convulsions (hazard ratio, 3.87; 95% CI, 1.35–12.8), and VPA monotherapy (hazard ratio, 3.86; 95% CI, 1.30–12.62) (Fig 3).

**Table 4:** Predictors of recurrent PSS from log-rank analysis.

Variable	*p* value
**Age, (<74)**	0.05
**Sex (male/female)**	0.66
** Cerebral infarction**	0.85
** Intracerebral hemorrhage**	0.47
***Localization of lesion***	
** Cortical**	0.029
** Frontal**	0.003
** Temporal**	0.72
** Parietal**	0.32
** Occipital**	0.91
** Traumatic brain injury**	0.39
** Neurosurgical intervention**	0.78
** Dementia**	0.42
** Previous seizure** ^**†**^	0.032
** mRS at discharge (3–5)**	0.031
***Stroke risk factors***	
** Hypertension**	0.27
** Diabetes mellitus**	0.09
** Dyslipidemia**	0.07
** Atrial fibrillation**	0.10
** Drinking alcohol**	0.93
** Smoking**	0.83
***EEG findings***	
** Focal or lateralized slowing**	0.46
** Epileptiform discharge**	0.72
***MRI findings***	
** DWI high intensity lesion after PSS**	0.69
**Modified Fazekas Scale (0-1/2-3)**	0.22
***Clinical features on admission***	
** Convulsion**	0.05
** Todd paralysis**	0.66
***Concomitant AEDs***	
** VPA monotherapy**	0.013
** CBZ monotherapy**	0.22
** PHT monotherapy**	0.15
** Polytherapy**	0.043
** None or discontinuation**	0.17

Abbreviations: PSS, post-stroke seizures; mRS, modified Rankin Scale; EEG, electroencephalogram; MRI, magnetic resonance imaging; DWI, diffusion weighted imaging; AEDs, antiepileptic drugs; VPA, valproic acid; CBZ, carbamazepine; PHT, phenytoin.

^†^Previous seizure: history of at least one episode of seizure when enrolled in this study.

## Discussion

This is the first study to identify predictors of recurrent PSS by considering both patient clinical characteristics and antiepileptic therapies. To date, numerous studies have examined risk factors and outcomes of PSS, but there has been little focus on risk factors for recurrence. We identified younger age, convulsions, and VPA monotherapy as significant independent risk factors for PSS recurrence.

Berges et al. reported recurrence in 54 of 94 patients (57.4%) exhibiting late PSS during a mean follow-up period of 47 months.[12] In this report, univariate analysis identified three factors associated with recurrent PSS, a hemorrhagic stroke component, poor Rankin scale score after the initial PSS episode, and occipital lesions, whereas multivariate analysis identified occipital lesions and a history of late seizure as independent risk factors. Thus, predictors of recurrent PSS did not necessarily correspond to predictors of early PSS, as neither hemorrhage nor poor Rankin scale score in the early post-stroke period predicted recurrence. However, their study did not examine associations between AED treatment regimens and frequency of recurrent PSS. In our study, we have found that VPA monotherapy was independently associated with PSS recurrence, although plasma concentrations had reached effective levels of VPA in 71.4% of patients (data not shown).

Most physicians agree that repeated PSS requires AED treatment as recommended by European guidelines,[18] but there is no consensus regarding the most effective class, dose, or duration for the prevention of recurrent PSS. In some countries, such as the United Kingdom, VPA remains a popular AED for the treatment of PSS.[19] In Japan, VPA is also a first-line AED for generalized epilepsy. According to the Cochrane review of AEDs for the primary and secondary prevention of PSS, the study by Gilad et al. (2007) was the only trial that has the high quality clinical evidence.[20] In this study, VPA monotherapy did not prevent the occurrence of late seizures compared with the placebo in patients with spontaneous, non-aneurysmal, intracerebral hemorrhage. Moreover, the choice of drug for elderly patients is often more complicated than that for younger patients because of the increased risk of side effects (due to lower hepatic and renal clearance in elderly patients) and the greater risk of drug interactions due to more frequent polypharmacy.[21, 22]

We demonstrated that younger age (<74 years old) was associated with recurrent PSS, consistent with the association of younger age with PSS or recurrent PSS reported in several previous studies.[23–27] One study reported a higher incidence of PSS in patients aged <65 years than in patients >85 years (10.7% vs. 1.6%; p < 0.001) based on 10-year estimates [24] The same study also identified hemorrhagic stroke and total anterior circulation infarction, clinical findings consistent with cortical location and greater stroke severity, as predictors of PSS. Another study found that the mean monthly seizure frequency was higher in younger elderly stroke patients (65–74 years) than in older patients (75–84 years) (7.8 ± 9.7 vs. 5.1 ± 5.1, p < 0.05), possibly because old patients require a lower AED dose.[27] Although we do not have sufficient data to assess the concentration of AEDs over a sufficient period of time, the higher incidence of recurrent PSS in the younger patients in our cohort may support a contribution of faster drug clearance and concomitant reduced plasma concentrations in seizure recurrence. The final independent predictor of recurrent PSS was the presence of convulsions. Convulsive seizures may promote epileptogenesis by inducing neural damage. However, we cannot exclude the possibility that increased risk of recurrence may also be explained by the fact that nonconvulsive seizures are not always detected at stroke presentation.[28].

The present study has several other limitations, including possible bias related to patient selection. First, only a relatively small number of subjects were analyzed because we could not gather complete outcome information for all study subjects. This may have caused the imbalance of sex; over two-third of patients in this study were males. The gender difference of stroke population may also have affected the imbalance. Previous studies showed that males have a higher prevalence of stroke in Japan.[29, 30] In fact, among 2420 stroke patients registered from January 2011 to July 2013, 1459 patients (60.3%) were males. Second, the chief diagnosis and complaint were only available from the database. In acute stroke cases, we may have missed some PSS patients. However, all previous admission records of the 104 patients in this study were rigorously reviewed. Moreover, the diagnosis of seizures at follow-up was based on patient reports and not confirmed by study physicians. Third, we recorded only the current AED regimen at final follow-up or recurrence and did not collect data on switching or suspending medications during the course of this study. Use of multivariate modeling may have reduced, but did not always eliminate, the effect of this on the results. Fourth, monotherapy using the second generation antiepileptic drugs had been officially unapproved in Japan during this study. So we could not obtain the sufficient data for discussing the efficacy of newer AEDs. Finally, it remains unclear to what extent future events (beyond approximately 3–18 months post-stroke) can be predicted by the identified factors because recurrent PSS data were collected at a single time point.

In conclusion, our study demonstrates that VPA monotherapy, younger age, and presence of convulsions are independent predictors of PSS recurrence. These results are hypothesis-generating and prospective randomized, double-blind studies are needed to assess the safest and most efficacious AEDs for the secondary prevention of PSS.
